# The relationship of genetic signature for cardiometabolic risk with biomarkers of inflammatory and oxidative stress in diabetic patients

**DOI:** 10.1186/s12902-025-01973-6

**Published:** 2025-07-01

**Authors:** Faezeh Abaj, Yasaman Aali, Fariba Najafi, Fariba Koohdani

**Affiliations:** 1 Department of Nutrition, Dietetics and Food, Faculty of Medicine, Nursing and Health Sciences, Monash University, Melbourne, Australia; 2 Victorian Heart Institute, Victoria Heart Hospital, Melbourne, Australia; 3 Department of Nutrition, Faculty of Medicine, Mashhad University of Medical Science, Mashhad, Iran; 4 Department of Sport Sciences and Physical Education, Mashhad Branch, Islamic Azad University, Mashhad, Iran; 5 Department of Cellular and Molecular Nutrition, School of Nutritional Sciences and Dietetics, Tehran University of Medical Sciences, PO Box: 141556117, Tehran, Iran

**Keywords:** Cardiometabolic risk factors, Type 2 diabetes mellitus (T2DM), Genetic risk score

## Abstract

The prevalence of cardiovascular diseases (CVDs) is increasing in most parts of the world. Several studies suggest that type 2 diabetes mellitus (T2DM) and CVD are induced by lifestyle behaviours and genetic factors. This study investigated the association between a genetic risk score (GRS) and cardio-metabolic risk factors among diabetic patients. The current cross-sectional study involved 700 diabetic patients. The genetic risk score was created by combining three single nucleotide polymorphisms [*Apolipoprotein A2 (APOA2)* (rs5082), *Ins/Del* (rs17240441) and *EcoR1polymorphism* (rs1042031) variants]. This polygenic risk score (PRS) was developed to predict cardiometabolic risks based on the presence of these common genetic variants. Standard protocols were used to measure anthropometric measurements and blood parameters. A significant association was observed between the GRS and several cardiometabolic risk factors, including BMI (β = 0.006, 95% CI = 0.001 to 0.01, *p* = 0.05) and WC (β = 0.006, 95% CI = 0.001 to 0.01, *p* = 0.02), in both crude and adjusted models. Additionally, a significant result was found between hs-CRP and GRS in the crude and adjusted models (β = 0.52, 95% CI = 0.2 to 0.83, *p* = 0.001). This study also revealed a reverse association between GRS and antioxidant markers such as PTX3 (β = -0.14, 95% CI= -0.23 to -0.04, *p* = 0.005), TAC (β = -0.02, 95% CI= -0.04 to < 0.001, *p* = 0.04), and SOD (β = -0.02, 95% CI= -0.04 to -0.006, *p* = 0.008). After controlling for confounding factors, the significant reverse associations between PTX3 (*P* = 0.009) and SOD (*P* = 0.009) with GRS were maintained. We found a significant positive association between GRS, including [*APOA2* (rs5082), *Ins/Del* (rs17240441) and *EcoR1* (rs1042031) variants] and cardiometabolic risk factors among T2DM patients.

## Introduction

Cardiovascular disease (CVD) accounts for one-third of all deaths worldwide, rendering it a major global health burden [1]. The risk factors for CVD are complex and include inflammation, dyslipidemia, high body mass index (BMI) and waist circumference (WC) [2, 3]. Key risk factors that place diabetic patients at a higher risk for CVDs include obesity, dyslipidemia and oxidative stress [4]. Although many factors contribute to the development of diabetes complications, strong evidence suggests that one of the primary causes of these issues is oxidative stress (OS), which is linked to pathological conditions such as obesity, diabetes, CVD, and atherogenic conditions [5, 6]. OS is a condition that arises from an imbalance between the body’s antioxidant system and reactive oxygen species (ROS) production [7, 8].

A strong predictor of CVDs has also been found to be genetic susceptibility [9, 10]. Large-scale genome-wide association studies (GWAS) have identified several novel genetic variants associated with obesity and CVD [11, 12]. Moreover, GRS, which is calculated by summing the risk alleles for each SNP in samples considerably smaller than those needed for GWAS, could be used to quickly assess the connections between genetic variables and CVD [13, 14]. *APOA2* is one of the key genes associated with an increased risk of obesity, T2DM, and CVDs. The second most prevalent protein on HDL-C particles, APOA2, is encoded by this gene [15, 16]. The antioxidant and reverse transport capacities of HDL-C appear to be impaired among participants carrying *APOA2* risk allele. Thus, increasing levels of APOA2 encourage atherosclerosis growth, which is a key indicator of CVDs [17]. One of the SNPs associated with anthropometric indices, obesity, and inflammatory markers is the *APOA2* (rs5082) [18].

The *Apo-B* gene, which is primarily involved in lipid homeostasis, has recently been implicated as a candidate for CVD risk in GWAS [19]. Based on a previous analysis, *Apo-B* was suggested to play a significant role in controlling lipid metabolism, as it is the most abundant protein in VLDL and LDL particles [20, 21]. Humans have polymorphic *Apo-B* genes; more frequently, *Apo-B* polymorphisms have been discovered for the *Ins/Del* (rs17240441) and *EcoR1* polymorphism (rs1042031) variants [22, 23]. According to prior studies, there are strong correlations between *Ins/Del and EcoR1* polymorphism and several CVD risk variables, including dyslipidemia, T2DM and inflammatory markers [24–26].

Therefore, it has been reported that each of the aforementioned genetic variants is individually linked to cardiometabolic risk factors in some populations [21, 27–29]. Given the following factors, the GRS is considered a useful tool for predicting cardiometabolic risk factors and increasing the power to detect them due to the combined impact of multiple SNPs. Moreover, according to the previously mentioned genetic factors, there is currently no evidence that cardio-metabolic traits and obesity-GRS are associated. To better identify diabetic patients at a higher risk of developing cardio-metabolic risk factors, the current research aimed to calculate the GRS through traits associated with obesity-related genetic markers, specifically *APOA2* (rs5082), *Ins/Del* (rs17240441), and *EcoR1* (rs1042031). These SNPs were chosen because they have a known functional impact, rather than being purely associative. One of the goals of this work was to develop something that could be easily tested in a practical way, so we focused on creating a score that could be assessed using a simple PCR test. This is much more accessible and cost-effective compared to more complex techniques like oligonucleotide chips or full genome sequencing. By focusing on functional variants, we hope to offer a risk score that could be used more widely in both clinical and population-based studies.

## Method

### Study population

The present study included 700 patients with T2DM referred from diabetes referral centers. In this current study, diabetic patients with fasting blood sugar levels above 126 mg/dl or those taking medications to lower glucose levels were included. Participants had no common ancestry and were unrelated.

Based on our previous larger study, all inclusion and exclusion criteria, demographics, physical activity (METs), and anthropometric measurements (BMI and WC) were collected. All protocols of this study were conducted in accordance with the Helsinki Declaration and approved by the Ethics Committee of Tehran University of Medical Sciences (IR.TUMS.VCR.REC.1395.15060). All participants completed a written informed consent form before participating in the study.

### Physical activity assessment

The International Physical Activity Questionnaire’s (IPAQ) short form was used to evaluate each participant’s level of physical activity during the previous week. IPAQ, a validated self-report questionnaire, was used to quantify physical activity over the last week [30].

### Biochemical assessments

Venous blood samples were collected from the study participants after a 12-hour fasting period. Total cholesterol (TC), high-density lipoprotein (HDL), low-density lipoprotein (LDL), and triglyceride (TG) levels were measured using an enzymatic colorimetric method (Pars Azmoon). Inflammatory markers- including C-reactive protein (CRP), interleukin-18 (IL-18), superoxide dismutase (SOD), prostaglandin F2 (PGF2), and pentraxin 3 (PTX3)- as well as total antioxidant capacity (TAC) were assessed with enzyme-linked immunosorbent assay kits (R&D Systems, Techne Corporation, Minneapolis, MN).

### DNA extraction, gene sequencing and GRS

The salting-out method was used to isolate DNA from whole blood. The *APOA2-265 T > C*, *Apo-B Ins/Del* (rs17240441), and *EcoR1* polymorphism (rs1042031) variations were genotyped using the polymerase chain reaction-restriction fragment length polymorphism (PCR-RFLP) approach. Electrophoresis of all products was performed in 8% agarose gel.

For *APOA2-265 T > C*, two pairs of primers—upstream primer 5′CAT GGG TTG ATA TGT CAGAGC-3′ and downstream primer 5′ TCA GGT GACAGG GAC TAT GG 3′—have been used to amplify the promoter region of the *APOA2* gene.

In addition, three genotype-containing fragments were identified (CC, TC, and TT). Primers F: (CACTGGGACCTACCAAGAG), R:(CTCGAAAGGAAGTGTAATCAC), and F: (5′CAGCTGGCGATGGACCCGCCGA3′), respectively, were used for PCR amplification of *EcoR1* polymorphism (rs1042031) and *Ins/Del* (rs17240441) for *ApoB* variants. Then, fragments with three genotypes for *EcoRI* (GG, AG, and AA) and *Ins/Del* (Ins/Ins, Ins/del, and del/del) were separated.

The *APOA2-265 T > C* (rs5082), EcoR1 polymorphism (rs1042031), and *Ins/Del* (rs17240441) polymorphisms, which have previously been linked to cardiometabolic indicators in GWAS and other studies, were combined to form the GRS. Each SNP was recoded as 0, 1, or 2 depending on the number of risk alleles associated with a higher BMI. Following that, the total number of risk alleles from the three SNPs was summed to determine the unweighted GRS. Each point on the GRS scale, ranging from 0 to 6, signifies a risk allele. Higher scores reflect a greater genetic susceptibility to increased BMI or body weight.

### Statistical analysis

The data’s normality was assessed using the Kolmogorov-Smirnov test. The overall features of the individuals were analyzed through descriptive statistics, including the mean, standard deviation, minimum, and maximum. Three groups of GRS scores were established: No risk (0 risk allele), moderate risk (1 risk allele), and high risk (> 2 risk alleles). The clinical features of the GRS groups were compared using ANOVA and ANCOVA in both crude and adjusted models. To evaluate the relationships between cardiometabolic risk variables (the dependent variable) and GRS, linear regression was employed in the crude and adjusted models. Age, physical activity, and calorie consumption were controlled for. SPSS version 25 (SPSS, Chicago, IL) was used for all statistical analyses, with a p-value lower than 0.05 considered statistically significant.

## Result

### Study population characteristics

A cross-sectional study involving 700 people with type 2 diabetes was conducted. Participants’ mean ages, weights, and BMI were 53.99 ± 6.55 years, 76.59 ± 14.01 kg, and 29.42 ± 4.7 kg/m², respectively. This study comprised 178 (38.9%) males and 280 (61.1%) females. A total of 218 individuals (39%) had more than two risk alleles, 240 (42.9.4%) had one risk allele, and 101 (18.1%) had no risk allele. In Table 1, the general characteristics of the research participants are described in greater detail.

**Table 1:** Characteristics of the study participants

Quantitative variables	Mean	SD	Minimum	Maximum
**Demographic characteristics and anthropometric**				
Age (year)	53.99	6.55	35	65
Body weight (kg)	76.59	14.01	46	133
BMI (kg/m^2^)	29.42	4.7	20	54
Waist Circumference (cm)	92.52	10.62	58.5	157
**Blood parameters**				
TC (mg/dl)	198.24	73.04	60	820
TG (mg/dl)	184.15	106.32	34	775
HDL-c (mg/dl)	53.32	12.65	17	145
LDL-c (mg/dl)	107.88	104	24	299
hs.CRP (mg/L)	2.22	1.51	0	5.54
PTX3(ng/ml)	2.62	0.46	0.92	4.43
IL18(pg/ml)	249.20	30.72	112.34	315.5
TAC(g/dl)	2.48	0.55	1.70	4.30
SOD(U/ml)	0.14	0.07	0.06	0.90
PGF2α(pg/ml)	72.43	6.13	56	87

SD: Standard deviation; BMI: Body mass index; WC: waist circumference; TC: Total cholesterol, TG: Triglyceride; LDL: Low density lipoprotein; HDL: High density lipoprotein; hs-CRP: High-sensitivity C-reactive protein; PTX3: Pentraxin 3; IL18: Interleukin-18; TAC: total antioxidant capacity; SOD: Superoxide dismutase; PGF2α: Prostaglandin F2α

### Difference in means of cardiometabolic variables across GRS

**Fig. 1:**
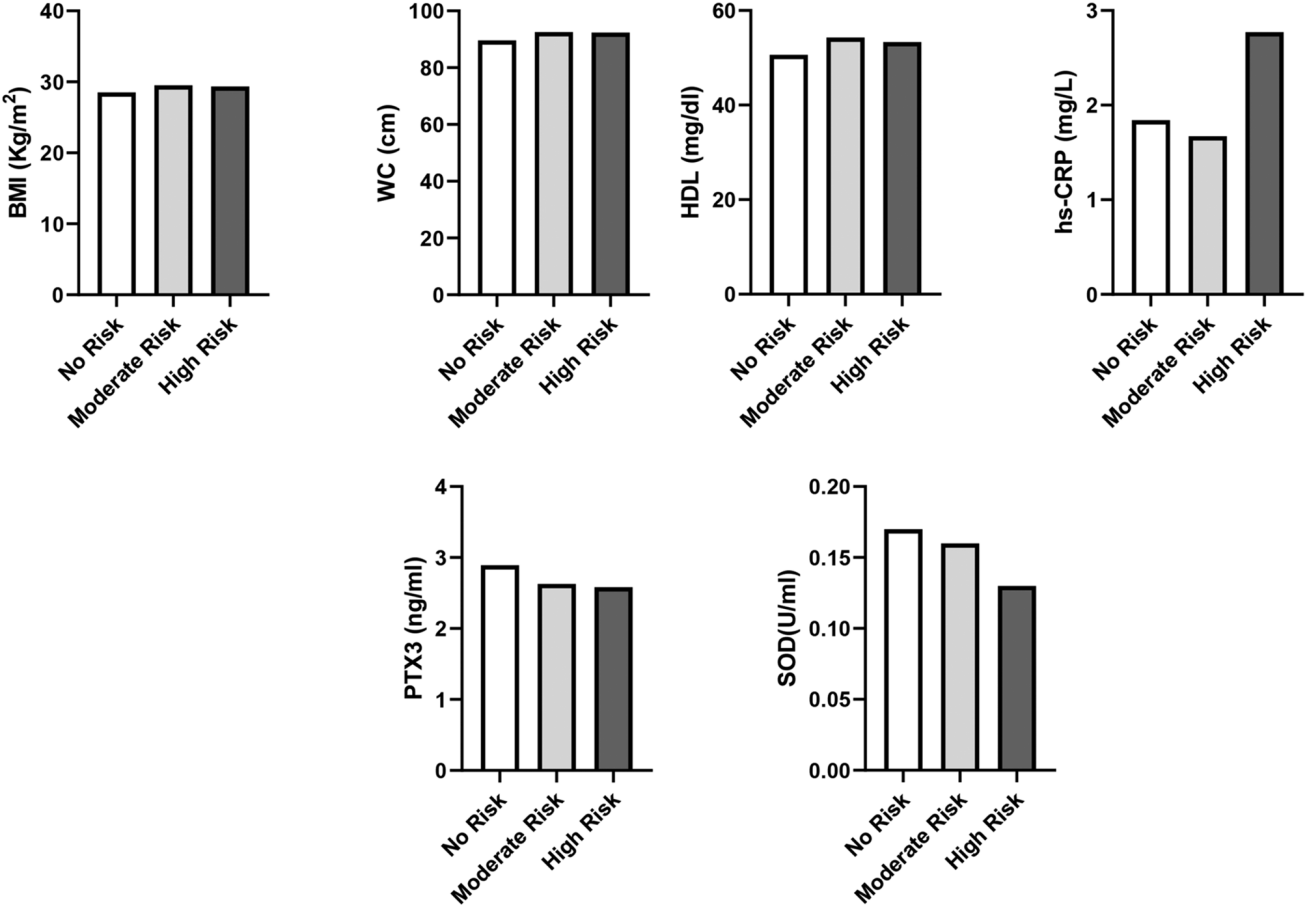
Difference in means of cardiometabolic variables across GRS groups. The genetic risk score was created by combining three single nucleotide polymorphisms [Apolipoprotein A2 (APOA2) (rs5082), Ins/Del (rs17240441) and EcoR1polymorphism (rs1042031) variants] No Risk=Without risk allele, Moderate Risk=1 risk allele and High Risk=>2 risk allele BMI - Body Mass Index, WC - Waist Circumference, HDL - High-Density Lipoprotein hs-CRP - High-Sensitivity C-Reactive Protein, PTX3 - Pentraxin 3, SOD-Superoxide Dismutase

Based on their genetic risk score, 700 patients were divided into different groups. Study variables were reported and compared across the genetic risk score, which was divided into three categories: No risk, moderate risk, and high risk (Table 2). Following classification, we discovered that patients at high risk for GRS had significantly higher BMI (*P* = 0.04), WC (*P* = 0.01), and hs-CRPs (*P* = 0.001). Antioxidant markers like PTX3 and SOD were significantly lower in patients with higher genetic risk scores (*P* = 0.02 and *P* = 0.01, respectively). Age, calorie consumption, and physical activity were adjusted for to preserve significant differences in BMI (*P* = 0.04), WC (*P* = 0.04), hs-CRP (*P* = 0.001), PTX3 (*P* = 0.01), and SOD (*P* = 0.03) (Fig. 1).

**Table 2:** The association between baseline characteristic and metabolic markers with GRS groups

	Genetic risk score				
Metabolic markers (*N*)	No risk (*N* = 101)	Moderate risk (*N* = 240)	High risk (*N* = 218)	*P*	*P* ^a^
	Without risk allele	1 risk allele	> 2 risk allele		
Sex (Male) N%	38 (17.6%)	94(43.5%)	84 (38.9%)	0.97	0.92
Age (year)	52.41 ± 6.13	52.76 ± 6.7	51.53 ± 6.94	0.83	0.85
Weight (Kg)	74.31 ± 12.64	76.97 ± 14.77	76.15 ± 12.71	0.26	0.23
BMI (kg/m^**2**^**)**	28.51 ± 4.71	29.52 ± 4.84	29.34 ± 4.39	**0.04**	0.09
WC (cm)	89.59 ± 10.03	92.54 ± 10.75	92.4 ± 10.98	**0.01**	0.24
Physical activity (Met.wk)	38.01 ± 6.35	37.81 ± 5.59	37.37 ± 5.15	0.55	**0.01**
HDL-c(mg/dl)	54.29 ± 11.99	53.32 ± 12.26	50.61 ± 9.65	**0.03**	0.43
LDL-c(mg/dl)	103.62 ± 35.37	110.06 ± 34.66	111.3 ± 36.66	0.12	0.92
CH (mg/dl)	196.21 ± 63.10	200.23 ± 61.66	191.62 ± 73.91	0.39	0.58
TG (mg/dl)	171.95 ± 93.87	179.59 ± 99.84	180.94 ± 106.88	0.76	0.24
hs.CRP (mg/L)	1.84 ± 1.31	1.67 ± 1.27	2.77 ± 1.57	**< 0.001**	**0.001**
PTX3 (ng/ml)	2.89 ± 0.46	2.63 ± 0.43	2.58 ± 0.45	**0.01**	**0.01**
IL18 (pg/ml)	245.37 ± 27.93	249.52 ± 29.82	251.3 ± 29.56	0.66	0.81
TAC (g/dl)	2.6 ± 0.5	2.46 ± 0.56	2.38 ± 0.56	0.2	0.14
SOD (U/ml)	0.17 ± 0.14	0.16 ± 0.05	0.13 ± 0.04	**0.02**	**0.03**
PGF2α (pg/ml)	70.73 ± 6.18	72.13 ± 5.49	73.19 ± 6.13	0.18	0.16

Data are presented as mean ± standard deviation (SD) or percent

Abbreviation: BMI: body mass index, HDL-c high density lipoprotein cholesterol, LDL-c = low density lipoprotein cholesterol, CH = cholesterol, TG =triglyceride, CRP = C-reactive protein, PTX3 = Pentraxin 3, IL18 = interleukin 18, TAC = total antioxidant capacity, SOD = superoxide dismutase, PGF2α = prostaglandinF2α

a Obtained from ANOVA, ANCOVA or Chi-square where appropriate

### Association of the GRS with cardiometabolic risk factors

The rough model demonstrated a significant correlation between the GRS and several anthropometric variables, including BMI (β = 0.006, 95% CI = 0.001 to 0.01, *p* = 0.05) and WC (β = 0.006, 95% CI = 0.001 to 0.01, *p* = 0.02) (Table 3). A positive direct relationship between BMI and WC was observed when accounting for confounding variables (age, calorie consumption, and physical activity) (*p* = 0.04).

**Table 3:** Association of GRS on cardiometabolic risk factors among

	GRS					
	Crude			Model 1		
	Β	95% CI	*P*-value	Β	95%CI	*P*-value
BMI (kg/m^2^)	0.006	< 0.001 to 0.01	**0.05**	0.007	0.001 to 0.01	**0.04**
WC (cm)	0.006	0.001 to 0.01	**0.02**	0.005	0.001 to 0.01	**0.04**
HDL-c (mg/dl)	0.96	-0.38 to 2.30	0.16	0.93	-0.38 to 2.24	0.16
LDL-c (mg/dl)	0.01	-0.002 to 0.03	0.08	0.01	-0.004 to 0.02	0.12
CH (mg/dl)	-3.37	-11.03 to 4.29	0.38	-4.04	-11.68 to 3.67	0.3
TG (mg/dl)	3.93	-7.85 to 15.72	0.51	3.48	-8.41 to 15.37	0.56
hs.CRP (mg/L)	0.55	0.23 to 0.87	**0.001**	0.52	0.2 to 0.83	**0.001**
PTX3 (ng/ml)	-0.14	-0.23 to -0.04	**0.005**	-0.14	-0.23 to -0.04	**0.006**
IL18 (pg/ml)	2.8	-3.47 to 9.08	0.37	3.05	-3.34 to 9.44	0.37
TAC (g/dl)	-0.02	-0.04 to < 0.001	**0.04**	-0.01	-0.03 to 0.001	**0.06**
SOD (U/ml)	-0.02	-0.04 to -0.006	**0.008**	-0.04	-0.07 to -0.01	**0.005**
PGF2α (pg/ml)	1.2	-0.09 to 2.5	**0.06**	0.007	-0.001 to 0.01	**0.06**

BMI: Body mass index; WC: waist circumference; TC: Total cholesterol, TG: Triglyceride; LDL: Low density lipoprotein; HDL: High density lipoprotein; hs-CRP: High-sensitivity C-reactive protein; PTX3: Pentraxin 3; IL18:Interleukin-18;TAC: total antioxidant capacity; SOD: Superoxide dismutase; PGF2α: Prostaglandin F2α GRS: Genetic risk score

† Calculated by linear regression

The findings revealed a significant positive relationship between hs-CRP and GRS in both crude (β = 0.55, 95% CI = 0.23 to 0.87, *p* = 0.001) and adjusted models (β = 0.52, 95% CI = 0.2 to 0.83, *p* = 0.001). This study also revealed a reverse association between GRS and antioxidant markers such as PTX3 (β = -0.14, 95% CI = -0.23 to -0.04, *p* = 0.005), TAC (β = -0.02, 95% CI = -0.04 to < 0.001, *p* = 0.04), and SOD (β = -0.02, 95% CI = -0.04 to -0.006, *p* = 0.008). After controlling for confounding factors, the significant reverse association between PTX3 (*P* = 0.009) and SOD (*P* = 0.009) with GRS was maintained. However, no significant association of the GRS with other metabolic variables such as TG, TC, LDL-C, HDL, and PGF2a was observed (Table 3).

## Discussion

The current cross-sectional study investigated the relationship between GRS and cardiometabolic risk factors, inflammation, and oxidative stress among 700 individuals with T2DM. The global prevalence of diabetes in adults (ages 20–79) was estimated to be 10.5% in 2021 and is projected to rise to 12.2% by 2045 [31]. The *APO B* and *APO A2* genes and their variants have been identified as potential candidates for individual susceptibility to disorders such as obesity, inflammation, and dyslipidemia [32, 33]. An increased ApoB: ApoA1 ratio is associated with a higher risk of atherosclerotic cardiovascular disease, regardless of LDL and HDL cholesterol levels [34].

The findings of this study indicate a positive association between GRS and anthropometric indices, such as BMI and WC. These results align with previous studies. In the 2020 study by Alsulami et al., participants with six or more risk alleles had a higher BMI than those with five or fewer risk alleles [35]. Similarly, Gholami et al. demonstrated a significant direct association between GRS and BMI and WC in a cohort of 391 overweight and obese women [36]. In the genome-wide interaction analysis, genetic variants are associated with BMI in participants under 50 [37].

*ApoB* rs512535 major G allele homozygotes were found to have an enhanced risk of metabolic syndrome (MetS) [38]. In another study, BMI was higher in participants with the Del allele than in those with the Ins allele [39]. Some studies showed results that were different from those of the present study. In the cross-sectional study, *Ins/Del* polymorphism (*Ins/Del*,* Del/Del*,* Ins/Ins*) and *EcoR1* polymorphism (*E−/E + and E−/E−*,* E+/E+*) were not related to anthropometric indices (BMI and WC) in patients with T2DM [40]. In the study by Rafiee et al., no significant association was found between the *ApoB Ins/Del* polymorphism, BMI, and WC [41]. There was no significant difference in BMI and WC between the *APO A2* polymorphism groups [42].

There was a positive association between GSR and hs.CRP and PGF2α, and there was also a negative association between GSR and PTX3, SOD, and TAC. These results were not consistent with the findings of some studies. In the cross-sectional study by Karimi et al., there was no association among EcoR1 polymorphism (*E-/E + and E−/E−*,* E+/E+*) and *Ins/Del* polymorphism (*Ins/Del*,* Del/Del*,* Ins/Ins*) with hs.CRP, SOD, and TAC in 700 patients with T2DM [40]. However, another study identified a significant positive association between hs-CRP and GRS in 391 overweight and obese women [36]. In the study by Mokhtary et al., TAC levels were considerably lower in *Del allele* carriers than in Ins/Ins homozygous individuals, while hs-CRP levels were significantly higher in obese Del allele carriers than in non-obese individuals [20].

A significant association was found between the CC genotype of *APO A2* and higher hs-CRP levels and lower PTX3 levels compared to *T allele* carriers [33]. The oxidative stress level of the *CC APO A2* genotype is higher than that of the *T allele* in T2DM patients [43].

*APOA2* level is associated with glucose, insulin, BMI and free fatty acids levels [18]. Hyperglycemia causes tissue damage through advanced glycation end products (AGEs). The glycation process affects circulating proteins, including serum albumin, lipoproteins, and insulin [44]. AGEs activate receptors like the receptor for AGEs (RAGE), which can lead to inflammation and oxidative stress [44]. The APOA-II levels can influence enzyme activity related to HDL-c (including types of paraoxonase), and one of their functions is to protect LDL-c from oxidation [45, 46]. The interaction of ABCA1 and Apo A1 induces signalling cascades involved in the anti-inflammatory pathway by ABCA1 [47]. In individuals with obesity, *Del allele* carriers are predisposed to oxidative stress, inflammation, and CVDs due to reduced antioxidant capacity and elevated inflammatory markers.

Dyslipidemia, stress, and inflammation are significant factors contributing to the complications of T2DM (including CVD) [48, 49]. There was an association between the heterozygous genotype of the XbaI *Apo B* gene and visceral obesity [50]. *Apo B* gene variations may influence fat deposition by transporting triglycerides (TG) to fat tissue via Apo B-containing very-low-density lipoprotein (VLDL) [51]. The reduction in the function and activity of the ATP-binding cassette transporter A1 (ABCA1) transporter was associated with increased adipose tissue [52]. There was an association between the *Del allele* and dyslipidemia in general obesity [41]. The liver’s APO B production is considered the main factor in VLDL secretion and levels of LDL-C and TG [53]. Also, the generation of *APO B* was regulated by posttranslational presecretory degradation through both lysosomal-dependent and proteasomal-dependent pathways [53]. Compared to Del/Del genotype, Ins allele carriers have higher VLDL levels in rat hepatoma cells [54].

The glycation process of LDL particles on ApoB can increase the extent of atherosclerosis, particularly in diabetic patients [55–57]. Reactive oxygen species (ROS) can activate Rho kinase, mitogen-activated protein kinase (MAPK), and tyrosine kinase, while inactivating tyrosine phosphatases, thereby modulating the expression and activation of pro-inflammatory genes [58–61]. Increasing blood sugar levels and fatty acids through NF-kb activation causes IL-1β activation. Pro-inflammatory cytokines and chemokines induce apoptosis and fibrosis in the pancreas, resulting in insulin disruption and systemic inflammation [62]. The disorder in the balance between antioxidant and oxidation activity leads to ROS generation and oxidative stress. Additionally, the interaction among oxidative stress, inflammation, and neurohumoral processes results in endothelial dysfunction, hypertrophy, and mitochondrial dysfunction [63].

This study has several strengths, particularly in exploring the relationship between *ApoB* and *ApoA* polymorphisms in diabetic patients. However, a few limitations should be acknowledged. First, due to the cross-sectional design, it is impossible to establish causal associations between the polymorphisms and outcomes. Secondly, the results may not be generalizable to the broader population, given the specific focus on diabetic patients.

While our polygenic risk score (PRS) provides useful insights based on these functional polymorphisms, we recognize that it has limitations. One important factor we did not include in our analysis is rare mutations—those with a minor allele frequency (MAF) less than 1%. Although these rare variants can have larger effects, their low frequency in the population makes them harder to capture statistically in large-scale studies. This is something we plan to consider in future work, as adding these rare mutations might improve the accuracy and predictive power of our PRS. Including them could also offer a more comprehensive understanding of the genetic factors at play in cardiometabolic risks, making our PRS even more useful in clinical settings.

## Conclusion

The present study demonstrates that in T2DM patients, polymorphisms of *APO B* and *APO A2* are associated with increased cardiometabolic factors. These findings emphasize the potential of GRS as a valuable tool for identifying individuals at higher risk. However, further research in diverse populations is essential to validate these results and explore the broader applicability of GRS in predicting cardiometabolic outcomes.

## Data Availability

The data are not publicly available because they contain the participants’ private information. Data are, however, available from the authors upon reasonable request.

## Abbreviations

T2DM: Type 2 Diabetes Mellitus
HDL: High-Density Lipoprotein Cholesterol
LDL: Low-Density Lipoprotein cholesterol
VLDL: Very Low-Density Lipoprotein
TG: Triglyceride
TC: Total Cholesterol
SNP: Single Nucleotide Polymorphism
